# Mycosis fungoides progression following COVID-19 mRNA vaccination: A case series

**DOI:** 10.1016/j.jdcr.2026.01.042

**Published:** 2026-02-03

**Authors:** Priyanka Kadam, Manasi Ladrigan, Brian Poligone

**Affiliations:** a Stony Brook Renaissance School of Medicine, Stony Brook, New York; b Comprehensive Dermatology of Rochester, PLLC, Rochester, New York; c Rochester Skin Lymphoma Medical Group, PLLC, Fairport, New York

**Keywords:** COVID-19 vaccination, CTCL, cutaneous t-cell lymphoma, general dermatology, MF, mycosis fungoides, oncology

## Introduction

Cutaneous T-cell lymphoma (CTCL) is a rare malignancy presenting with patches, plaques, or tumors.^1^ We report 5 long-term CTCL patients who experienced disease progression following COVID-19 vaccination. Each patient had documented disease stability prior to vaccination. The literature demonstrates that COVID-19 vaccination is safe and effective,^2^^,^^3^ yet prior reports describe CTCL onset or relapse after vaccination, including after a first dose.^4^ While our retrospective series cannot establish causation, and we maintain the importance of vaccination against COVID-19, we seek to contribute to the emerging literature on this observation. In this series, we describe the temporal relationship, clinical presentation, management, and outcomes in these patients.

## Methods

We included biopsy-confirmed CTCL with stable disease/partial remission before vaccination who then deteriorated (new tumors, large-cell transformation, systemic spread, or death). Data were derived from records, pathology, imaging, and treatment logs; all patients provided consent.

### Case 1 presentation

**Fig 1:**
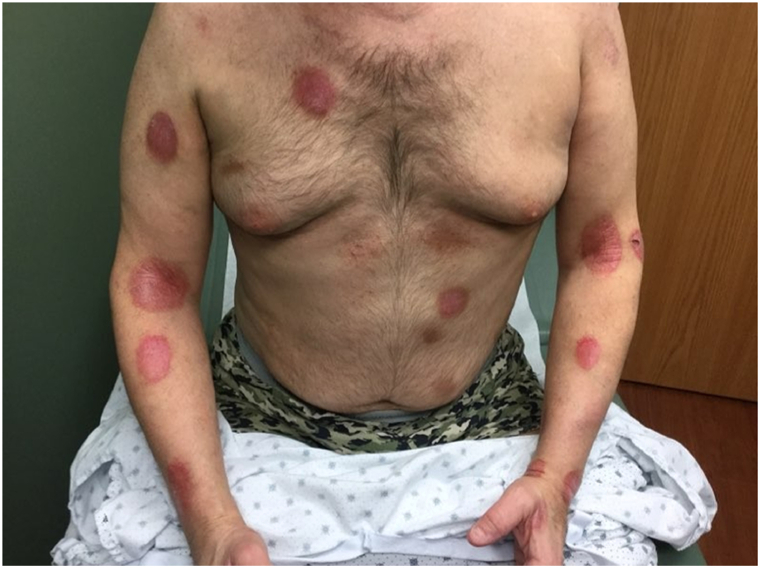
Patient 1: Clinical presentation of recurrent patches and plaques without evidence of tumors follow stem cell transplant.

Patient 1 was a 53-year-old male with mycosis fungoides (MF) diagnosed in 2014 (T2N0M0B0). After progression to tumors with large cell transformation, he received a stem cell transplant in February 2015. One-year post-transplant (Fig 1), he had recurrence of patches and plaques without tumors, managed with topical steroids, nitrogen mustard, PUVA, 5-fluorouracil, bexarotene, brentuximab, and interferon-alpha.

**Fig 2:**
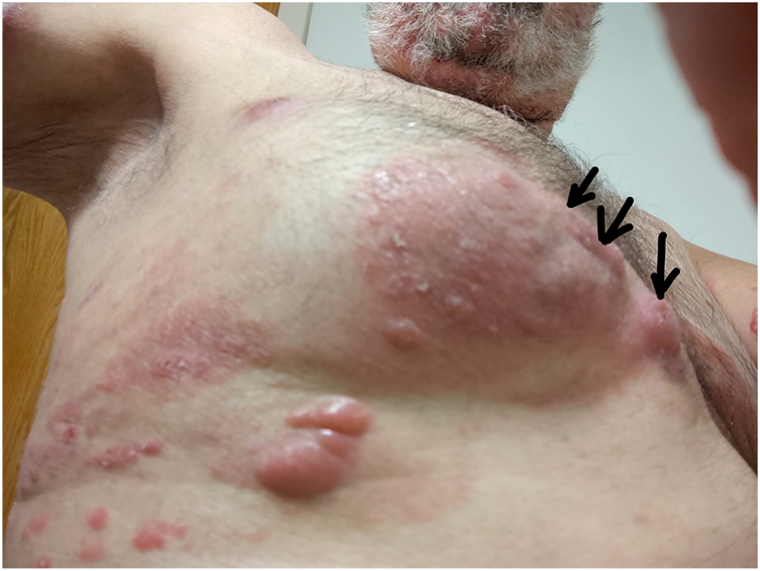
Patient 1: Clinical evidence of disease progression characterized by the multiple cutaneous tumors. *Black arrows* indicate new tumors.

By early 2021 he had patch and plaque disease without tumors that had remained stable for years. Following vaccination in Feb-Mar 2021, the plaques thickened and new tumors developed by April 2021. Despite multiple lines of therapy, including anti-CD30 re-challenge, clinical trial cellular inhibitor of apoptosis protein inhibitor, photopheresis, histone deacetylase inhibitors, focal radiotherapy, and later chemotherapy, he developed widely scattered tumors (Fig 2) and nodal/pleural involvement on imaging. He unfortunately passed away on August 17, 2023 (Table I).

**Table I:** Clinical timeline and objective disease metrics

Patient	Age/sex	CTCL subtype	Disease duration prevaccination	Prevaccination disease status	Vaccination date	Time to symptom progression following vaccination	Key progression events	Date of death (mo, y)
1	53/M	MF (post-SCT)	7 y (dx 2014)	Stable plaque disease without tumors ×6 y post-SCT in 2015; stable on multiple therapies	Feb-Mar 2021	Weeks (new tumors reported by April 2021)	New tumor formation within weeks of vaccination; nodal/pleural spread; treatment-refractory	August, 2023
2	76/F	MF	8 y (dx 2013)	Stage IB, stable on triamcinolone (last visit Aug 2019)	Oct 13, 2021	3 d	New tumors 3d postvax; ulceration; subQ/nodal spread	September, 2023
3	50/M	Folliculotropic MF	11 y (dx 2010)	Stable on TNM and topical corticosteroids (Jan 2021 visit)	Feb 2021	Months (Patient-reported “soon after” Feb 2021 vaccination); documented (Jan 2022)∗	Widespread flare; LCT; pancreatic mass; visceral mets	September, 2023
4†	63/M	MF (h/o CD30+ ALCL)	8 y (dx 2013)	Partially controlled on pralatrexate (Oct 2020 visit)	Dec 2020-Jan 2021	Weeks (new tumors requiring RT by Jan 2021)	New tumors requiring RT; LCT (May 2021); pulmonary metastases	October, 2021
5‡	63/M	MF (new onset)	N/A	No prior skin disease	April 2021	Weeks (symptoms began within a mo of vaccination (see Fig 3))	New onset MF with LCT at diagnosis; refractory to multiple lines; 2 SCTs	Currently alive

Summary of prevaccination disease status, vaccination timing, and objective evidence of disease acceleration for each patient.

*LCT, large cell transformation;MF*, Mycosis fungoides; *TNM*, nitrogen mustard.

∗ Patient 3's documented follow-up gap (11 months) reflects patient self-management; he consistently attributed symptom onset to his February 2021 vaccination.

† Patient 4 had partially controlled (not fully stable) disease at vaccination, with mixed response to pralatrexate. However, subsequent progression was accelerated with rapid development of LCT and pulmonary metastases.

‡ Patient 5 represents new-onset disease rather than progression, included to illustrate de novo CTCL with immediate transformation.

### Case 2 presentation

Patient 2 was a 76-year-old female with stage IB MF (T2N0M0B0) diagnosed in 2013, stable on triamcinolone. She reported that 3 days after she received the COVID-19 vaccinations in October 2021, she noticed many new pruritic plaques and large papules, which progressed to tumors over the following weeks. She treated her advancing disease between the end of 2021 and September 2023 with various therapies, including oral bexarotene with topical bexarotenes, phototherapy, total skin electron beam therapy, suberoylanilide hydroxamic acid, gemcitabine, brentuximab and bendamustine. Ultimately, she elected comfort-focused care and passed away on September 4, 2023 (Table I).

### Case 3 presentation

Patient 3 was a 50-year-old man with folliculotropic MF (T1N0M0B0) diagnosed in 2010. After total skin electron beam radiation in 2014, he maintained good disease control on nitrogen mustard and topical steroids. Plaques recurring in 2017 were well-managed with the same regimen. His disease remained stable on this minimal maintenance regimen for over 3 years, with his last pre-vaccination visit in January 2021 showing well-controlled plaque disease. He received the COVID-19 mRNA vaccination series in February 2021. The patient did not return for follow-up as he had young children and tried to manage his worsening disease independently. At the January 2022 visit, he reported significant disease flaring with new, thickened plaques on the chest, arms, legs, and lower back. The patient specifically noted the progression began after his COVID-19 vaccination in February 2021. He described his disease, which had previously been always stable (even prior to his total skin electron beam), as now “different” and “aggressively spreading.” He was initially started on methotrexate.

Beginning February 2023, he began spot radiation, interferon alpha, and discontinued methotrexate use. During his June 2023 visit, his tumors had spread to his right temple, chest, and upper thighs, and a PET scan showed a pancreatic mass and pelvic/inguinal lymphadenopathy. Pathology of a subcutaneous chest mass showed transformed large cell MF. He began pralatrexate infusions in the beginning of July 2023 and began planning chemotherapy and a stem cell transplant. Despite treatment, computed tomography scan in August 2023 revealed further progression with new pulmonary nodules, peritoneal disease, and pancreatic enlargement. He was hospitalized in August 2023, almost septic, and received cyclophosphamide, hydroxydaunorubicin (doxorubicin), oncovin (vincristine), etoposide, and prednisone chemotherapy for 2 days. During a hospital visit prior to his death, the patient again stated that his disease progression began after his COVID-19 vaccinations. He passed away on September 27, 2023 (Table I).

### Case 4 presentation

Patient 4 was a 63-year-old male with history of CD30+ anaplastic T-cell lymphoma (diagnosed in 2000, treated with radiation) who developed CD30-negative MF plaques in 2013. He achieved remissions during recurring plaques in 2013 and 2016 with bexarotene and brentuximab, and was maintained on bexarotene and phototherapy.

A 2018 plaque recurrence responded to localized radiation. A staging PET scan in late October 2018 showed no lymph node or organ involvement. Flow cytometry was normal. He subsequently received mogamulizumab and investigational BNZ-1 for his plaque disease as plaques were leading to skin breakdown and discomfort. Although he had a partial response with BNZ-1, he switched in early October 2020 to pralatrexate, which was also partially effective. He received the COVID-19 vaccine between his December 28, 2020 visit and January 29, 2021 visit.

By January 2021, he developed tumors of the left anterior chest wall, left anterior ankle, left posterior ankle, and left posterior knee. These were treated in January 2021 with 1200 cGy of total body irradiation. Given his rapid progression, he began the AST-660 clinical trial of tolinapant, a cellular inhibitor of apoptosis protein inhibitor, and continued to receive radiotherapy. Biopsy of a new tumor in May 2021 showed large cell transformation of his MF. A computed tomography scan from July, 2021 showed a new mass in the right lung and several smaller ones on the left lung. The patient started romidepsin and a biopsy of the lung showed lymphoma. The patient chose to proceed to home hospice and passed away in October 2021 (Table I).

### Case 5 presentation

**Fig 3:**
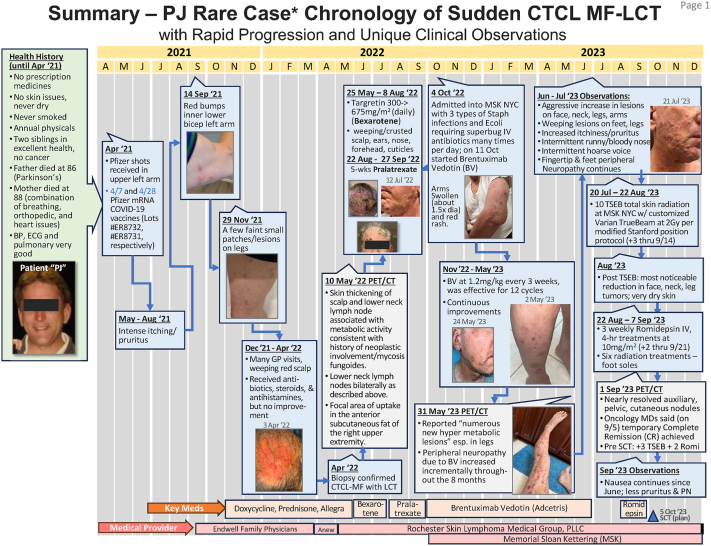
Patient 5: Clinical timeline from the time of vaccination documenting disease progression until his first stem cell transplant.

This patient had a new onset diagnosis of MF. A 63-year-old male presented in May 2022 with a chief complaint of a rash located on his left scalp, left forehead, right hand, and scalp. His lesions began about 1 year prior, following COVID-19 vaccination in May 2021; he had no prior skin symptoms (Fig 3). He initially tried topical and oral steroids and oral antibiotics, which reduced his itching but not the skin eruption. Multiple skin biopsies were taken in April 2022, and 4 out 5 of the biopsies showed mycosis fungoides, with the scalp showing transformed mycosis fungoides.

This patient was started on oral bexarotene, pralatrexate, and brentuximab but continued to progress with tumors. Repeat skin biopsy from June 2023 demonstrated MF with large cell transformation. In October 2023, he had a stem cell transplant. In December 2023, roughly 2 years after COVID-19 vaccinations, he exhibited recurrence on the skin. He was enrolled in a clinical trial of a CDK9 inhibitor but progressed quickly with multiple new lesions. He completed a chimeric antigen receptor T-cell CD5 cell treatment, which also was unsuccessful. He restarted romidepsin in October 2024. His disease remains significant yet stable and a second stem cell transplant was completed March 2025 (Table I).

## Discussion

CTCL is a rare, chronic malignancy of skin-homing T-cells with variable progression. Four patients had stable disease prior to vaccination but subsequently experienced transformation to large cell lymphoma or systemic involvement. Patient 5 developed new onset MF with large cell transformation within 1 year of vaccination. As a major center for cutaneous lymphoma treatment, we recognize these 5 patients as notable outliers. While our findings do not establish causality, they warrant further investigation.

The interval from vaccination to documented clinical progression varied from days (Case 2) to weeks (Cases 1, 4) to months (Cases 3, 5), consistent with the published literature (Table I).^4^^,^^5^ Case 3’s first documented follow-up occurred 11 months postvaccination. However, this patient previously had over a decade of indolent disease controlled with topical therapy alone, and he consistently attributed his disease flare to his vaccination throughout his clinical course. His subsequent trajectory, progressing from stable plaque disease to transformed large cell lymphoma with visceral metastases within 2.5 years, contrasted with his prior stable course. The variability in progression may reflect differences in baseline tumor burden, individual immune response kinetics, or thresholds for seeking clinical evaluation.

Prior literature showing progression of CTCL after the COVID-19 vaccine often describes lymphomatoid papulosis, a self-resolving condition (33% of cases in Olszewska et al^5^). In contrast, our series describes aggressive MF with large cell transformation and systemic spread; 4 out of 5 patients died. This distinction is clinically important. Whereas lymphomatoid papulosis flares may represent transient immune activation without long-term consequences, our patients developed treatment-refractory disease. Etesami et al identified MF in only 11 of 71 drug- and vaccine-associated CTCL cases (15.5%), suggesting that true MF progression with large cell transformation remains underreported.^4^

While we cannot establish whether the observed temporal relationship reflects causation, we explored different biological mechanisms that could link vaccination to disease progression. We emphasize that these remain speculative and require experimental validation. mRNA-based vaccines, elicit a robust immune response characterized by activation of T-cells and proinflammatory cytokine release. Chronic antigenic stimulation (from a vaccine) may lead to upregulation of checkpoint markers such as programmed cell death protein-1.^6^^,^^7^ This might suggest the use of checkpoint inhibitors in these patients. mRNA vaccines elicit a strong Th1 response (interferon-gamma, IL-2) and this may disrupt the cytokine balance in MF patients.^6^ Lastly, the proinflammatory cytokine storm that is induced by the vaccine, may induce a compensatory Th2 response in the patient (IL-4, IL-5, IL-10, and IL-13), accelerating disease progression.^8^ In our patients, these changes were not transient but resulted in progressive disease and, in 4 cases, death. We are aware of at least 6 other cases of MF or Sezary syndrome associated with COVID-19 vaccination in the literature,^9^^,^^10^ plus additional MF-like lymphoproliferative reactions.^11^ Olszewska et al reviewed 24 CTCL cases following vaccination,^5^ and other reports document relapse of primary cutaneous anaplastic large cell lymphoma, Sézary syndrome, folliculotropic MF, and early-stage MF with lymphomatoid papulosis.^12^

However, alternative explanations are plausible. CTCL exhibits unpredictable progression, and transformation to aggressive disease occurs spontaneously without identified triggers. The long disease duration in our patients may have placed them at natural risk for transformation independent of vaccination. The heterogeneous timing of progression (days to months) lacks the consistency expected of a uniform biological mechanism. All possibilities require prospective investigation. The possibility that these cases represent coincidental timing cannot be dismissed. Our case series captures only patients with adverse outcomes and cannot account for CTCL patients who remained stable or improved after vaccination.

Our observations align with reports of systemic lymphomas following COVID-19 vaccination, including non-Hodgkin lymphoma cases within days of mRNA vaccination.^13^^,^^14^ Gambichler et al reported spontaneous pcALCL regression postvaccination,^15^ suggesting that vaccine-induced immune activation can exacerbate or suppress cancer. Clarifying these mechanisms will help us determine whether certain patients face increased risk after vaccination.

We emphasize that COVID-19 vaccination remains crucial for public health. Our findings do not imply patients with CTCL should avoid vaccination but suggest heightened vigilance and close monitoring post-vaccination.

Critical limitations preclude causal inference. As an uncontrolled retrospective case series, we cannot establish causation or account for selection bias; patients who experienced disease stability or improvement after vaccination would not have been captured. CTCL has heterogeneous trajectories, and prior therapies or tumor genomics may explain progression independent of vaccination. Retrospective chart abstraction and patient recall introduce bias; larger prospective cohorts with standardized staging and molecular profiling would help clarify these findings.

## Conclusion

We report 5 CTCL patients who experienced disease progression following COVID-19 vaccination. While the temporal proximity is notable, multiple competing explanations exist, and our retrospective design cannot distinguish causation from coincidence. These observations may represent either a rare vaccine-associated complication in a susceptible subset of patients or the expected natural disease progression occurring by chance.

Despite these cases, COVID-19 vaccination remains essential for public health, and our findings should not discourage vaccination in CTCL patients. If clinicians choose to discuss these observations with patients, such conversations should emphasize the unproven nature of any risk and the established benefits of vaccination. Prospective controlled studies are urgently needed to determine whether these observations reflect a true association and quantify absolute risk to enable evidence-based care.

## Conflicts of interest

None disclosed.
